# Anaerobic benzene mineralization by natural microbial communities from Niger Delta

**DOI:** 10.1007/s10532-020-09922-x

**Published:** 2020-12-02

**Authors:** Samuel C. Eziuzor, Matthias Schmidt, Carsten Vogt

**Affiliations:** Department of Isotope Biogeochemistry, Helmholtz Centre for Environmental Research-UFZ, Permoserstraße 15, 04318 Leipzig, Germany

**Keywords:** Benzene, Niger delta, Anaerobic hydrocarbon degradation, Biodiversity, Iron reduction

## Abstract

The Niger Delta is one of the most damaged ecosystems in the world, mainly due to petroleum contamination by oil exploration accidents. We investigated the natural attenuation potential of Niger Delta subsurface sediment samples for anaerobic hydrocarbon degradation using benzene as a model compound under iron-reducing, sulfate-reducing, and methanogenic conditions. Benzene was slowly mineralized under methanogenic and iron-reducing conditions using nitrilotriacetic acid (NTA)-Fe(III), or poorly crystalline Fe(III) oxyhydroxides as electron acceptors, analyzed by measurement of ^13^CO_2_ produced from added ^13^C-labelled benzene. Highest mineralization rates were observed in microcosms amended with Fe(III) oxyhydroxides. The microbial communities of benzene-mineralizing enrichment cultures were characterized by next-generation sequencing of the genes coding for 16S rRNA and methyl coenzyme M reductase A (mcrA). Abundant phylotypes were affiliated to Betaproteobacteriales, Ignavibacteriales, Desulfuromonadales, and Methanosarcinales of the genera Methanosarcina and Methanothrix, illustrating that the enriched benzene-mineralizing communities were diverse and may contain more than a single benzene degrader. The diversity of the microbial communities was furthermore confirmed by scanning helium-ion microscopy which revealed the presence of various rod-shaped as well as filamentous microbial morphotypes.

**Electronic supplementary material:**

The online version of this article (10.1007/s10532-020-09922-x) contains supplementary material, which is available to authorized users.

## Introduction

The Niger Delta is one of the world’s most important wetland and coastal marine ecosystem consisting of rain forest, mangroves and fresh water swamps. Oil exploration activities resulted in massive contaminations with petroleum compounds, rendering the Niger Delta region into one of the five most severely damaged ecosystems in the world (Kadafa 2012; Linden and Palsson 2013; UNEP 2011). There is urgent and vital need for a sustainable clean-up and restoration of the natural resources in order to save for the future and human health. In the long term, natural attenuation of these compounds may support remediation of the contaminated land; however, the potential of the indigenous microbial community to degrade oil compounds at anoxic conditions has not been determined yet in the Niger Delta area. Principally, several petroleum components can be mineralized under oxic or anoxic conditions (Head et al. 2008; Jones et al. 2008). In water-saturated soils and sediments, severe contamination by hydrocarbons quickly renders conditions from oxic to anoxic due to rapid consumption of oxygen by aerobic hydrocarbon degraders. Hence, although anaerobic biodegradation is considerably slower than aerobic biodegradation, it is the more relevant natural attenuation mechanism at petroleum contaminated areas (Meckenstock et al. 2015). Whereas sulfate is the principal electron acceptor for anaerobic hydrocarbon degradation in marine sediments due to its high concentration in seawater (≈ 28 mM), ferric iron (Fe(III)) is thought to be the counterpart at terrestrial sites due to the ubiquitous presence of iron in terrestrial sediments (Ehrlich 1996). Consequently, Fe(III) has been found to be a crucial electron acceptor for degradation of organic compounds in freshwater systems (Lovley 2006), e.g. shallow aquifers (Essaid et al. 2011; Lovley and Philip 1986; Meckenstock et al. 2015). However, in contrast to soluble electron acceptors for anaerobic respiration like sulfate or nitrate, iron is insoluble at most environmental conditions and forms with OH or O several oxides, e.g., Fe(III) oxyhydroxides (goethite, ferrihydrite) or mixed-valent iron minerals (magnetite) (Usman et al. 2018). Thus, it is rather complicated to enrich for Fe(III)-reducing microorganisms due to the multitude of different environmental forms of Fe(III); for example, many pure cultures reduce poorly crystalline Fe(III) oxyhydroxides better than highly crystalline Fe(III) oxyhydroxides (Lovley 2006). An alternative method for culturing iron reducers in the laboratory is to apply soluble Fe(III) in form of Fe(III)-citrate or Fe(III) chelated with nitrilotriacetic acid (Fe(III)-NTA), since some cultures have been shown to grow much faster with soluble Fe(III) compared to poorly crystalline Fe(III) (Lovely 2006). Additionally, iron metabolizing bacteria exhibit characteristic organic structures and crystalline Fe minerals (Joens et al. 2013; Bryne et al. 2018). Besides sulfate and Fe(III), carbonate is a relevant electron acceptor for hydrocarbon degradation, resulting in syntrophic consortia of fermenters and methanogens (Jiménez et al. 2016).

Benzene is a priority pollutant at hydrocarbon-polluted environments due to its abundance, toxicity and relatively high water solubility; notably, it is far more persistent under anoxic conditions compared to alkylated monoaromatics (Vogt et al. 2011), making benzene an important model compound for natural attenuation microcosm studies with regard to petroleum or gasoline contaminations. A couple of enrichment cultures have been described mineralizing benzene under sulfate-reducing (Lovley et al. 1995; Phelps et al. 1996; Vogt et al. 2007; Musat and Widdel 2008; Laban et al. 2009), methanogenic (Weiner and Lovley 1998; Ulrich and Edwards 2003; Sakai et al. 2009), nitrate-reducing (Burland and Edwards 1999; Ulrich and Edwards 2003; Keller et al. 2018; van der Zaan et al. 2012) or iron-reducing (Lovely et al. 1996; Kazumi et al. 1997; Jahn et al. 2005; Botton and Parsons 2006; Kunipali et al. 2007) conditions. Key organisms characterized in some of these cultures were found to be diverse (summarized in Vogt et al. 2011), indicating that phylotypes belonging to several different taxa are able to metabolize benzene under anoxic conditions. It was also observed that benzene was syntrophically mineralized under several electron-acceptor conditions; phylotypes affiliated to the Gram-positive Peptococcaceae were shown to be key benzene degraders (Kunipali et al. 2007; Laban et al. 2009; Herrmann et al. 2010; van der Zaan et al. 2012; Luo et al. 2014). Only a few benzene degrading isolates have been described either using nitrate (Coates et al. 2001; Kasai et al. 2006) or iron (Holmes et al. 2011; Zhang et al. 2012) as electron acceptor. The initial step of benzene activation in the absence of oxygen is still under debate.

The goal of this study was to elucidate the potential of Niger Delta sediment for anaerobic hydrocarbon natural attenuation processes using benzene as model compound. We focused on iron-reducing conditions as Fe(III) is expected to be a major electron acceptor in the investigated sediments. Amendment of ^13^C-labelled benzene to the cultures and analyzing the ^13^CO_2_ δ-values allowed for determining the low mineralization rates which are below the detection limits of standard protocols. This study presents first results on the anaerobic hydrocarbon degradation potential of Niger Delta sediments and will contribute to the knowledge of the variety of anaerobic benzene degraders under iron-reducing conditions.

## Materials and methods

### Chemicals

Chemicals were purchased from Fluka (Steinheim, Germany), Merck (Darmstadt, Germany), Roth (Karlsruhe, Germany) and Sigma-Aldrich (Taufkirchen, Germany) in p.a. quality if not otherwise stated. Benzene- ^13^C_6_ was purchased from Sigma-Aldrich with an isotopic purity of 99 atom % ^13^C.

### Site description and sampling procedure

The sediments used in this study originated from Ogoni, a region in the Niger Delta, Nigeria, which is rich in hydrocarbon and natural gas resources but massively contaminated due to oil production related activities. Samples were obtained from three points per sites in Gokana (4.6577° N 7.2980° E, 4.6560° N 7.2769° E, 4.6559° N 7.2767° E) and Tai (4.7008° N 7.2781° E, 4.7007° N 7.2782° E, 4.7009° N 7.2793° E) and pulled together as a sample from each site on 12 June, 2015, and from two points per site in Eleme (4.8536° N 7.0689° E, 4.7493° 7.2458° E), and one point only from site in Khana (4.6290° N 7.4558° E) on 11 July, 2015. The points representing each site were combined as a representative of the site.

Sediment samples were taken from depths of approximately 0.3 m as a wet, hydrocarbon contaminated samples and immediately transferred into sterile plastic bags. The filled bags were transferred into an anaerobic jar including a reduction kit (AnaeroGen, Oxoid Ltd, Basingstoke, UK) to remove remaining oxygen. Samples were stored at 4 °C in the dark until further processing.

### Setup of enrichment cultures

All enrichment cultures were set up in an anaerobic glove box (Coy Laboratory Products Inc., Grass Lake, USA) containing a gas atmosphere of 95% N_2_ and 5% H_2._ Precisely, 25 g sediment samples (wet weight) were transferred into 240 mL serum bottles (Glasgerätebau Ochs, Bovenden-Lenglern, Germany). Subsequently, microcosms were filled up with anoxic carbonate buffered mineral salt medium (Vogt et al. 2007) to a total volume of 200 mL. Four variants of mineral salt medium were prepared: (i) no amendments, (ii) addition of anoxic Na_2_SO_4_ (1 M) to a final concentration of 20 mM sulfate, (iii) addition of amorphous Fe(III) oxyhydroxide (prepared according to Lovely, 2006) to a final concentration of 100 mM, (iv) addition of Fe(III) NTA (Lovely 2006) to a final concentration of 100 mM. The non-amended mineral salt medium contained only bicarbonate (30 mM) and sulfate (1.7 mM) as electron acceptors and was designated to select for carbonate-reducing methanogens. Initial sediment enrichment cultures were spiked with a mixture of the non-labeled model hydrocarbons benzene, ethylbenzene (each in a final concentration of 100 µM), and naphthalene (final concentration ~ 100 µM) dissolved in acetone (2 mL of acetone) to monitor the potential for hydrocarbon degradation at anoxic conditions. For each sediment and electron acceptor, two replicate bottles were prepared. The bottles were closed gastight with aluminum crimped Teflon-coated butyl septa (ESWE Analysentechnik, Gera, Germany), and incubated statically at room temperature in the dark.

After one year of incubation, suspensions from the initial Khana sediment enrichment cultures were transferred into fresh mineral salt medium amended with ^13^C-labeled benzene to ascertain mineralization of benzene by determining the production of ^13^CO_2_. For this, 120 mL serum bottles (Glasgerätebau Ochs, Bovenden-Lendern, Germany) were filled with 90 mL mineral salt medium and 10 mL enrichment culture. Similar to the initial enrichment cultures, four types of electron acceptor conditions were set up: 20 mM sulfate, 100 mM amorphous Fe(III) oxyhydroxide, 100 mM Fe(III) NTA, or carbonate buffer (30 mM) and 1.7 mM sulfate for methanogenic cultures. The bottles were closed gastight with aluminum crimped Teflon-coated butyl septa and spiked thereafter with ^13^C-labeled benzene (99%) using glass syringes (Hamilton Company, USA) to reach concentrations of 100 µM in each bottle. Subsequently, the bottles were incubated for approximately 25 months statically at room temperature in the dark. Benzene, sulfate and Fe(II) concentrations in the respective bottles were regularly analyzed. In addition, abiotic control bottles containing only mineral salt medium and ^13^C-labeled benzene (99%) in concentrations of around 100 µM were setup for each electron acceptor condition. After 1 year of incubation, a second transfer was made from iron-reducing cultures (which used amorphous Fe(III) oxyhydroxide or Fe(III) NTA as electron acceptors) into fresh medium amended with 100 µM ^13^C-labeled benzene and amorphous Fe(III) oxyhydroxide or goethite, respectively, prepared according to Lovely (2006) (Fig. S1).

Samples for chemical or microbial analyses were always taken by sterile syringes previously flushed with nitrogen to exclude oxygen contaminations inside the microcosms.

### Chemical and microscopic analyses

Sulfide concentrations were determined spectrophotometrically by the method of Cline as described elsewhere (Herrmann et al. 2008). Ferrous iron concentrations were determined spectrophotometrically by a modified Ferrozine method (Stookey 1970; Viollier et al. 2000).

Benzene concentrations in the microcosms were determined by a gas chromatograph equipped with a flame ionization detector (GC-FID) as described by Keller and colleagues (2018).

The carbon isotope ratios of produced CO_2_ and CH_4_ were determined using a gas chromatograph-isotope ratio mass spectrometer (GC-IRMS) as described elsewhere (Herrmann et al. 2010). Carbon isotope ratios were expressed in the delta notation in per mil (δ ^13^C/‰) units relative to the Vienna Pee Dee Belemite (VPDB) according to the following equation (Coplen 2011):

$$ \delta^{{{13}}} {\text{C}}_{{{\text{sample}}}} \left[ \permille \right] \, = \, \left( {{\text{R}}_{{{\text{sample}}}} /{\text{R}}_{{{\text{reference}}}} {-}{ 1}} \right) $$

where R_sample_ and R_reference_ are the ratios of the heavy isotope to the light isotope (^13^C/^12^C) in the sample and in the standard (VPDB).

Mineralization rates were calculated using the equation stated in Dorer et al. (2016); the procedure is described in detail in the supporting information (S1).

In order to investigate microbial morphotypes and mineralized iron, samples were taken at the end of experiment from enrichment cultures incubated under iron-reducing conditions amended with Fe(III) NTA as iron source. After filtration, chemical fixation, dehydration and drying the samples were investigated by scanning helium ion microscopy (HIM) (Joens et al. 2013; Byrne et al. 2018) as described in detail in the supporting information, S2.

### Microbial community analysis

#### Extraction of DNA

DNA was extracted from sediment samples and enrichment cultures. Ten gram of sediment sample was taken to extract DNA using the DNeasy PowerMax Soil Kit (Qiagen Inc, Germany) according to manufacturer’s instructions. Extracted DNA was concentrated using Amicon Ultra-0.5-mL centrifugal filters (Merck KGaA, Germany) applying the protocol given by the manufacturer. DNA from the enrichment cultures were extracted as follows: In microcosms amended with Fe(III) species, 50 mL of the liquid phase was centrifuged at 4 °C and 10,000×*g* for 10 min to obtain pellets. In microcosms incubated at sulfate-reducing and methanogenic conditions, cells were concentrated by filtering approximately 60 mL liquid phase using a 0.2 µm filter membrane (Merck KGaA, Germany); the filter was subsequently cut into slices with a sterile scalpel. DNA was extracted from both the pellets or filter slices using a DNeasy PowerSoil Kit (Qiagen Inc, Germany) according to manufacturer’s instructions except that DNA was eluted in 60 µL final volume instead of 100 µL to obtain a more concentrated DNA.

DNA quality and quantification was done using a NanoDrop ND 1000 spectral photometer (Thermo Fisher Scientific, United States) and a Qubit fluorometer using the Qubit dsDNA BR assay kit (Thermo Fisher Scientific GmbH, Dreieich, Germany). Extracted DNA was stored at − 20 °C until further use.

#### Amplicon sequencing of the genes for 16S rRNA and mcrA

The microbial communities of selected enrichment cultures were analyzed by amplicon sequencing of genes coding for 16S rRNA genes of bacteria and methyl coenzyme M reductase (mcrA) for methanogenic archaea. Prior to amplicon sequencing, the V3-V4 regions of 16S rRNA genes of bacteria were PCR-amplified using the primers 341f (CCTACGGGNGGGCWGCAG) and 785r (GACTACHVGGGTATCTAAKCC) according to Klindworth et al. (2013). mcrA genes were PCR-amplified using the primers mlas (GGTGGTGTMGGDTTCACMCARTA) and mcrA-rev (CGTTCATBGCGTAGTTVGGRTAGT) according to Steinberg and Regan (2008). Amplicon sequencing was carried out using an Illumina MiSeq platform (Illumina, CA, USA) generating 2 × 250 bp paired-end reads provided by the Department of Environmental Microbiology, Helmholtz Centre for Environmental Research-UFZ. The PCR products were purified to remove leftover primers using AMpure XP beads and then used for an indexing PCR. Sequencing libraries were prepared according to manufacturer recommendations. The resulting de-multiplexed raw sequences were further processed using QIIME 2 (version 2019.1; https://qiime2.org; Caporaso et al. 2010). The following workflow was applied: (i) FastQC quality filtering to remove low quality reads, (ii) PHRED-based filtering, (iii) cutadapt to remove primer sequences, (iv) DADA2 to de-noise, trim, de-replicate, merge paired-end reads, remove chimeras, and infer exact amplicon sequencing variants (ASVs), and (v) taxonomic assignment using a pre-trained QIIME2 compatible SILVA database (release 132). The bioinformatics workflow was similar for the 16S rRNA and mcrA genes except that for mcrA genes, the taxonomic assignment was done using a customized database created by downloading all mcrA sequences from the Functional Gene Repository containing taxonomic information to at least the genus level (Fish et al. 2013). The ASVs with less than 1% relative abundance across the samples were excluded from further data analysis. The demultiplexed raw sequence reads were submitted to the National Center for Biotechnology Information, NCBI Sequence Reads Archive with the accession number PRJNA580489 (https://www.ncbi.nlm.nih.gov/sra/PRJNA580489).

## Results and discussion

### Mineralization of benzene at different electron acceptor conditions

**Fig. 1:**
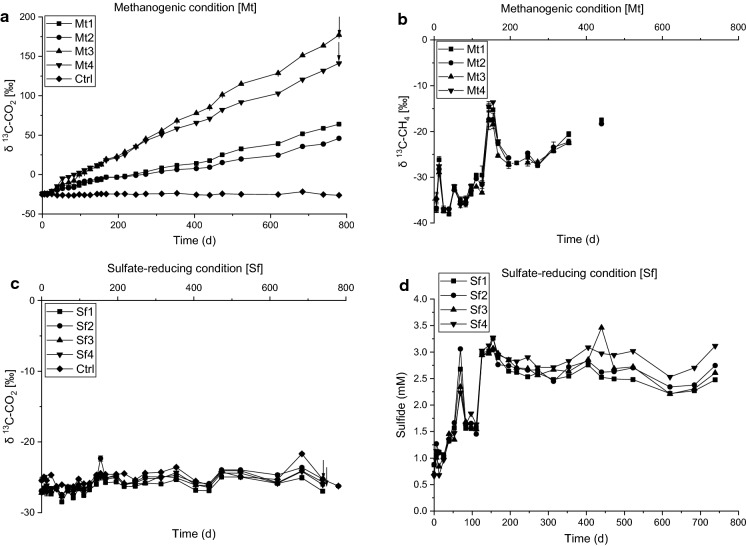
Mineralization of ^13^C-labeled benzene to ^13^C-CO_2_ (**a**) and ^13^C-CH_4_ (**b**) under methanogenic condition [Mt], and ^13^C-CO_2_ (c) production and sulfide concentrations (**d**) under sulfate-reducing condition [Sf] of the first transfer enrichment cultures. Arrows indicate cultures used for amplicon sequencing and time point of sampling. In abiotic controls (Ctrl), methane was not detected throughout the incubation time, and sulfide was not analyzed

**Fig. 2:**
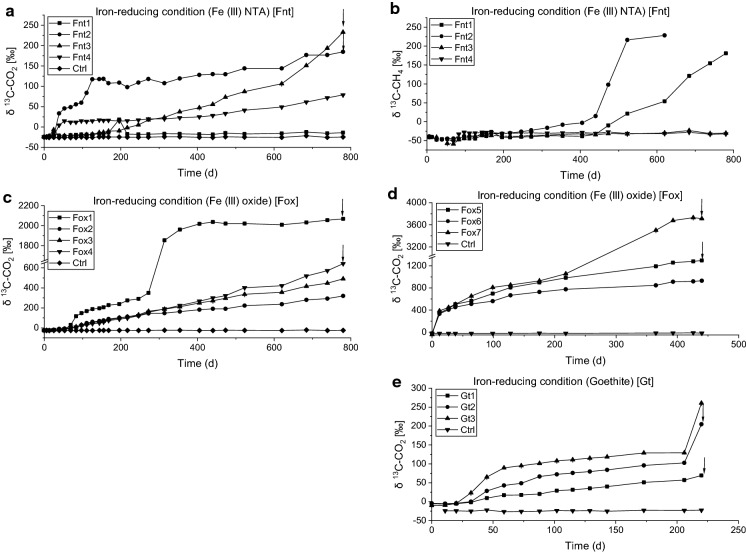
Mineralization of ^13^C-labeled benzene (i) to ^13^C-CO_2_ (a) and ^13^C-CH_4_ (b) with Fe(III) NTA as electron acceptor [Fnt] by the first transferred enrichment culture, (ii) to ^13^C-CO_2_ (c, d) with Fe(III) oxide as electron acceptor by first [Fox1–4] and second [Fox5–7] transferred cultures, respectively, and (iii) to ^13^C-CO_2_ with goethite as electron acceptor by second transferred Fnt2 enrichment culture (see Supplementary Fig. S2 for details of the enrichment procedure). The sharp increase of ^13^C-CO_2_ production in microcosm Fox1 was linked to the time point of culture transfer to setup for second transfer [Fox5–7] between 280 to 320 days. Arrows indicate cultures used for amplicon sequencing and time point of sampling

In initial sediment microcosms from four investigated sites (Gokana, Tai, Eleme, Khana), the rate of degradation of ethylbenzene, naphthalene and benzene was low under iron-reducing, sulfate-reducing and methanogenic conditions even after 365 days of incubation (data not shown). Low degradation rates for certain hydrocarbons under anoxic conditions-resulting from lag phases of months to years- can be due to a very small initial number of degraders in a sample. In addition, growth rates and growth yields can be low due to small energy amounts conserved during substrate oxidation, as reported e.g. for methyl *tert*-butyl ether (MTBE) (Somsamak et al. 2001) or naphthalene (Galushko et al. 1999). For benzene, it was repeatedly observed that the compound is not degraded under various anoxic conditions in distinct sediments samples (summarized by Vogt et al. 2011), raising the question whether degradation was too slow to be detected by analyzing benzene removal or whether benzene degraders were actually absent. Thus, to clarify this question, we applied a more sensitive approach to detect anaerobic benzene degradation in the sediment samples by spiking the microcosms with ^13^C-labeled benzene and analyzing the release of ^13^CO_2_ and ^13^CH_4_ via GC-IRMS. This technique has already been successfully used to verify mineralization of apparently persistent hydrocarbons under anoxic conditions (Morasch et al. 2007; Nijenhuis et al. 2007). Intending also to enrich anaerobic benzene degraders in the microcosms by sequential dilution, new cultures were set up at iron-reducing, sulfate-reducing and methanogenic conditions by transferring 10% of the initial enrichment culture in mineral salt media which were spiked with ^13^C-labeled benzene. For this, we used the Khana sediment enrichment cultures, which showed slightly higher degradation rates compared to the microcosms prepared from sediment of the other three studied sites (data not shown). The time course of δ^13^C-CO_2_ values of these enrichment cultures are shown in Figs. 1a, c and 2a, c, d, e. Enrichments amended with soluble or solid Fe(III) as well as enrichments containing mainly bicarbonate as electron acceptors mineralized benzene. The mineralization rates differed between replicates as well as between electron-acceptor conditions; highest rates (up to 1.47 µM day^−1^) were observed with solid Fe(III) as electron acceptor (Tables 1, S1). The observed rates are similar to benzene oxidation rates previously reported for aquifer sediment or groundwater microcosms at iron-reducing conditions (Table 1).

**Table 1:** Overview of laboratory studies with regard to benzene degradation under iron-reducing conditions

Art of study	Inoculum	Fe(III) source	Benzene degradation rate	Proof of mineralization	Community structure	References
Laboratory microcosms	Sediment of a hydrocarbon-contaminated aquifer	Natural sediments, in some cases addition of amorphous Fe(III) oxyhydroxide: added chelating agents (NTA, EDTA, ethanol diglycine, humic acids) stimulated benzene degradation	n.d	Stoichiometry of degraded benzene to produced Fe(II) corresponded to theoretical ratio for benzene mineralization with Fe(III) [30: 1]; Production of ^14^CO_2_ (86%) out of ^14^C-benzene	n.d	Lovley et al. (1994), Lovley et al. (1996)
Laboratory microcosms	Sediment of a oil refinery site	No additional Fe(III) source; Fe(III) reduction could not be sustained after reduction of naturally available Fe(III); cultures switched to degrading benzene via sulfate reduction or methanogenesis	Not available	Analysis of the stoichiometry of degraded benzene to produced Fe(II)	n.d	Nales et al. (1998), Ulrich et al. (2003)
Laboratory microcosms	Freshwater or estuarine sediment, polluted or non-polluted	Amorphous Fe(III) oxyhydroxide	Freshwater sediment: ca. 1 µM day^−1^; Estuarine sediment: ca. 0.65 µM day^−1^, could not be repeated	n.d	n.d	Kazumi et al. (1997)
Laboratory microcosms	Sediments of hydrocarbon-contaminated aquifers	No additional Fe(III) source, sediments were thought to contain Fe(III) as main electron acceptor	Not reported; two out of six sediments mineralized benzene	Production of ^14^CO_2_ (10% and 50%) out of ^14^C-benzene	Enrichment of *Geobacteriaceae* correlated to anaerobic benzene oxidation	Anderson et al. (1998)
Laboratory microcosms	Hydrocarbon-polluted or non-polluted sediments	Amorphous Fe(III) oxyhydroxide	No benzene degradation within 161 days of incubation – but benzene degradation occurred with other electron acceptors	n.d	n.d	Phelps and Young (1999)
Laboratory enrichment cultures & aquifer sediment	Sediment of hydrocarbon-contaminated aquifer	Amorphous Fe(III) oxyhydroxide + 4 mM NTA as Fe (III) chelator + 1.3 mM Fe(II) chloride as a reductant	n.d	Microcosms mineralized ^14^C-benzene to ^14^C-CO_2_ (25% in 25 days)	Enrichment of *Geobacter* in benzene-degrading sediment and Fe(III)-reducing microcosms	Rooney-Varga et al. (1999)
Laboratory microcosms	Groundwater and sediment of a hydrocarbon-contaminated aquifer	Amorphous Fe(III) oxyhydroxide	Groundwater: 0.16–0.34 µM day^−1^. Sediment: 0.09 µM day^−1^	Determined by analysis of degraded benzene and produced Fe(II)	*Geobacter* dominant member of the microcosm communities	Botton and Parsons (2006, 2007), Botton et al. (2007)
Laboratory microcosms	Sediment of river Rhine	Amorphous Fe(III) oxyhydroxide	0.12–0.29 µM day^−1^	Stoichiometric amount of 70% soluble Fe(II) was produced per mol of benzene degraded	n.d	Villatoro et al. (2008a, b)
Laboratory microcosms	Sludge from groundwater monitoring well, hydrocarbon-contaminated aquifer	Amorphous Fe(III) oxyhydroxide ± AQDS, resin Amberlite XAD7 as benzene carrier phase	n.d	Stoichiometry of degraded benzene to produced Fe(II) corresponded to theoretical ratio for benzene mineralization with Fe(III)—electron recoveries of 74% to 82% ferric iron	n.d	Jahn et al. (2005)
Enrichment culture	Soil of former coal gasification site	Amorphous Fe(III) oxyhydroxide	n.d	Around 85% electron recovery from Fe(III) reduction; stoichiometric production of ^13^C-CO_2_ from ^13^C-benzene	Gram-positive Peptococcaceae as main benzene assimilating phylotype; putative syntrophy with Deltaproteobacterium (Desulfobulbus) and Actinobacteria	Kunapuli et al. (2007)
Pure culture	*Ferroglobus placidus* (hyperthermophilic)	Amorphous Fe(III) oxyhydroxide	n.d	Stoichiometry of iron reduction and benzene oxidation close to the theoretical value expected for mineralization of benzene by iron(III) reduction	*Ferroglobus placidus*	Holmes et al. (2011)
Pure cultures	*Geobacter metallireducens Geobacter* sp. strain Ben	Amorphous Fe(III) oxyhydroxide (strain Ben) or Fe(III) citrate (*G. metallireducens*)	n.d	Mineralization of ^14^C-benzene to ^14^C-CO_2_; reduction of Fe(III) to Fe(II); cell growth linked to benzene degradation and iron reduction	*Geobacter metallireducens, Geobacter* sp. strain Ben	Zhang et al. (2012)
Laboratory microcosms	Hydrocarbon-contaminated sediment	Amorphous Fe(III) oxyhydroxide	0.025–0.496 µM day^−1^	Analysis of δ^13^CO_2_ from ^13^C-labeled benzene	Betaproteobacteriales, Ignavibacteriales dominant phylotypes	This study
Laboratory microcosms	Hydrocarbon-contaminated sediment	Fe(III) nitrilotriacetic acid	0.001–0.019 µM day^−1^	Analysis of δ^13^CO_2_ from ^13^C-labeled benzene	Ignavibacteriales, Thermodesulfovibrionia, Thalassobaculales dominant phylotypes	This study
Laboratory microcosms	Hydrocarbon-contaminated sediment	Goethite	0.019–0.069 µM day^−1^	Analysis of δ^13^CO_2_ from ^13^C-labeled benzene	Desulfuromonadales dominant phylotypes	This study

*n.d.* not determined

In microcosms containing bicarbonate as main electron acceptor, produced methane became only slightly enriched in ^13^C upon incubation time (up to − 17 ‰ δ^13^C-CH_4_; Fig. 1b), indicating that the formation of methane was mainly due to the reduction of non-labeled substrates, e.g. bicarbonate from the bicarbonate buffer of the mineral salt medium, and not from ^13^C-labeled intermediates of benzene oxidation, e.g. acetate. Notably, methane considerably enriched in ^13^C (more than + 200 ‰ δ^13^C-CH_4_) was detected in two of four replicate cultures amended with Fe(III)-NTA (Fig. 2b), indicating that labeled metabolites of anaerobic benzene degradation or compounds from recycled labelled necromass were used as electron donors by methanogens in these enrichment cultures.

Benzene was not mineralized under sulfate-reducing conditions within 780 days in four replicate cultures (Fig. 1c), although cultures produced 2–3 mM sulfide during incubation (Fig. 1d), showing that the inoculum contained considerable amounts of electron donors, probably organic compounds of the original sediment usable for sulfate reduction. We cannot exclude that benzene oxidation under sulfate-reducing conditions was inhibited by elevated concentrations of sulfide as observed before in sulfidic groundwater enrichment cultures (Jin et al. 2007).

### Microbial community structure at different electron-acceptor conditions

Amplicon sequencing of the 16S rRNA and mcrA genes were carried out to determine abundant phylotypes of the microbial communities in the sediment and in each of the enriched cultures depending on the selected electron acceptor conditions, to get indications which phylotypes might be involved in benzene mineralization. The original sediments contained eubacterial phylotypes belonging mainly to Acidobacteria and several classes of Proteobacteria (Alpha-, Beta-, Delta-, and Gammaproteobacteria) (Fig. S2). In particular, a relatively high abundance of Bacteriodales, Holophagales and Betaproteobacteriales were found in the Khana sediment samples. Major identified methanogenic taxa in the original sediments were Methanocellales, Methanobacteriales and Methanosarcinales (Fig. S2).

**Fig. 3:**
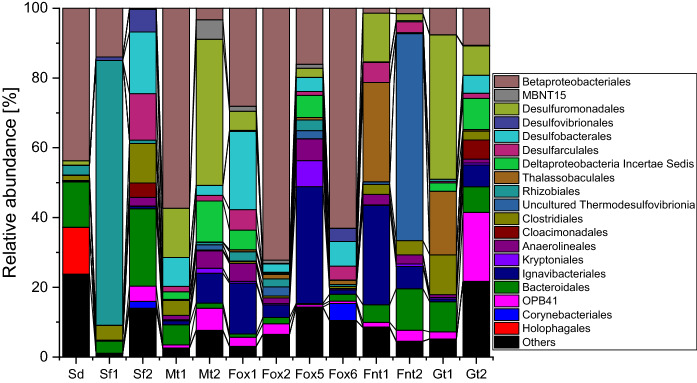
Bacterial community compositions based on 16S rRNA gene sequencing. First transfer enrichment cultures include Sf1, Sf2, Mt1, Mt2, Fox1, Fox2, Fnt1, Fnt2, while second transfer enrichment cultures are Fox5, Fox6, Gt1, Gt2. *Sd* original sediment, *Sf* sulfate-reducing condition, *Fox* iron-reducing condition with Fe(III) oxide, *Fnt* iron-reducing condition with Fe(III) NTA; *Mt* methanogenic condition, *Gt* iron-reducing condition with goethite

The three selected electron acceptor conditions (methanogenic, sulfate-reducing, iron-reducing) caused a shift to different microbial communities; notably, different communities were also observed depending on the used source of Fe(III) as electron acceptor (Fig. 3).

Microcosms amended with bicarbonate as main electron acceptor mineralized benzene rather slowly compared to other previously described benzene-degrading enrichment cultures (Tables 1; S1); however, the slight increase in δ^13^C values of methane showed that benzene was at least partly mineralized by methanogens. The analyzed cultures were dominated by Betaproteobacteriales and several Deltaproteobacteria, especially Desulfuromonadales and Desulfobacterales; also Ignavibacteriales and phylotypes belonging to OPB-41 were present in higher abundance. The methanogenic community was dominated by phylotypes belonging to Methanosarcina. Methanogens have not been described yet for primary degradation of aromatics; usually, degradation of aromatics or other more complex hydrocarbons at methanogenic conditions is carried out by fermenting organisms in syntrophic interaction with methanogens, the latter consuming fermentations products like hydrogen or acetate (Jiménez et al. 2016). Members of the Methanosarcina are capable of using hydrogen, acetate and other low weight organics as electron donors for methanogenesis, hence being metabolically more versatile than other methanogenic taxa and thus ideal partners for potential syntrophic interactions with hydrocarbon-fermenting partner organisms (Jiménez et al. 2016). Deltaproteobacteria have frequently been reported as benzene consumers at methanogenic conditions: the Deltaproteobacterium ORM-2 related to the Syntrophobacterales (Luo et al. 2016) and a phlyotype belonging to the Desulfobacterales (Sakai et al. 2009; Noguchi et al. 2014). Desulfobacterales-related phylotypes have also been suggested as benzene-consuming organisms under sulfate-reducing conditions (Oka et al. 2008; Phelps et al. 1998; Musat and Widdel 2008). In addition, Desulfuromonadales belonging to the family Geobacteriaceae have been described in a couple of studies as benzene degraders at iron-reducing conditions (summarized in Table 1 and discussion below); however, they have not been reported for benzene mineralization with other electron acceptors. Betaproteobacteriales have been suggested to be involved in benzene degradation at nitrate-reducing conditions (Kasai et al. 2006; Luo et al. 2014), but have not been observed in methanogenic benzene-degrading enrichment cultures.

Cultures amended with amorphous Fe(III) oxyhydroxide showed the highest benzene mineralization rates observed in this study. Two replicate cultures (Fox1, Fox2) were dominated after the first transfer by similar taxa in slightly different abundance: Betaproteobacteriales, Ignavibacteriales, and Desulfobacterales were the most abundant. A similar pattern was seen after the second transfer (Fox5, Fox6). Notably, in two cultures (Fox2, Fox6), the microbial community comprised more than 50% of Betaproteobacteriales, and in one culture (Fox5), around 33% of Ignavibacteriales. Two replicate cultures amended with Fe(III) NTA both comprised of Desulfuromonadales, Desulfarculales, Clostridiales, Anaerolineales, Ignavibacteriales, Bacteroidales and phylotypes belonging to OPB-41; notably, the abundance of Betaproteobacteriales was below 2%. However, Thalassobaculales were highly enriched only in culture Fnt1 and absent in culture Fnt2, whereas phylotypes belonging to the Thermodesulfovibrionia were highly enriched (> 50% abundance) in culture Fnt2 and absent in culture Fnt1. Investigation of Fe(III)-reducing bacteria with HIM revealed the presence of both crystalline Fe materials as well as rod-shaped prokaryotes; in addition rather long, filamentous prokaryotic organisms were observed (Fig. S3). The study is limited in analyzing further the organic structures and the identity of the observed filamentous prokaryotes found in Fe(III) amended cultures.

**Fig. 4:**
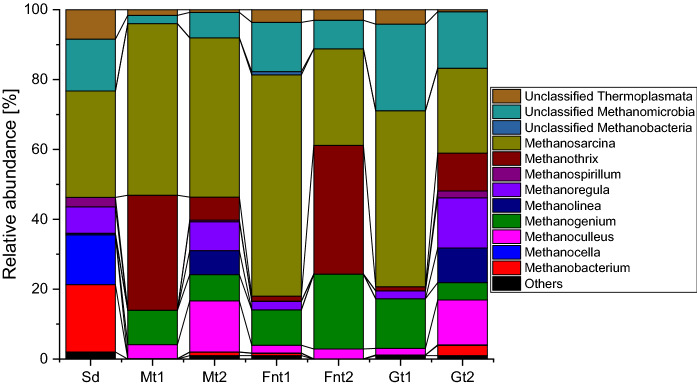
Methanogenic community compositions based on sequencing of mcrA genes. *Sd* original sediment, *Fnt* iron-reducing condition with Fe(III) NTA, *Mt* methanogenic condition, *Gt* iron-reducing condition with goethite

One culture incubated with Fe(III) NTA (Fnt2) was transferred and amended with goethite as electron acceptor. In these cultures (Gt1, Gt2), the number of Betaproteobacteriales, Desulfuromonadales, Desulfobacterales and phylotypes belonging to OPB-41 increased, whereas the previously observed Thermodesulfovibrionia disappeared in Gt2. The cultures with the chelated iron mineral showed relative abundance of Methanosarcinales; specifically the genus Methanosarcina (28–63% relative abundance), Methanothrix, and a Methanomicrobiales of the genus Methanogenium (Fig. 4).

Phylotypes belonging to the family Geobacteriaceae of the order Desulfuromonadales have been repeatedly observed to be key players of benzene degradation at iron-reducing conditions. They were detected in higher abundance in benzene-mineralizing laboratory microcosms containing sediments of hydrocarbon-contaminated aquifers as inoculum and amended with amorphous Fe(III) oxyhydroxide as electron acceptor (Rooney-Varga et al. 1999; Botton and Parsons 2006, 2007; Botton et al. 2007). Higher abundance of Geobacteriaceae were also oberved in benzene-mineralizing sediments containing Fe(III) as main natural electron acceptor (Anderson et al. 1998). Notably, also pure cultures of Geobacter strains have been described for benzene degradation under iron-reducing conditions (Zhang et al. 2012). Holmes et al. (2011) reported the use of hyperthermophilic *Ferroglobus placidus* as a pure culture with amorphous Fe(III) oxyhydroxide in benzene oxidation. Besides Geobacteriaceae, a phylotype belonging to the Peptococcaceae was described as main benzene-fermenting organisms in an enrichment culture prepared form soil of a former coal gasification site and amended with amorphous Fe(III) oxyhydroxide as electron acceptor, in putative syntrophy with phylotypes belonging to Desulfobulbus and Actinobacteria (Kunapuli et al. 2007). In our study, Ignavibacteriales and Betaproteobacteriales were identified as highly abundant phylotypes in benzene-mineralizing enrichment cultures amended with Fe(III) oxyhydroxide, indicating growth upon benzene consumption. Notably, Ignavibacteria were recently suggested to be responsible for the opening of the benzene ring in an anaerobic 2,4,6-trichlorophenol-degrading enrichment culture (Song et al. 2019); in addition, they were observed in higher abundance in a biofilm covering an anode of continuous-flow bioelectrical system applied for BTEX degradation (Palma et al. 2019). Several Betaproteobacteriales are known as degraders of aromatic hydrocarbons using nitrate as electron acceptor including benzene (Vogt et al. 2011; Weelink et al. 2010). Notably, some of them were shown to be able to use ferric iron as electron acceptor for aromatics hydrocarbon mineralization (Dorer et al. 2016). This assertion highlights that iron and nitrate reducers occupy similar ecological niches.

However, due to the putative availability of (non-labelled) background organic compounds, the observed low mineralization rates and the associated relatively low amounts of benzene mineralized in the single cultures (between 1 and 218 µM, Table SI 1), it might be that the benzene mineralizing organisms were of minor abundance in the analyzed communities. Phylotypes belonging to the Peptococcaceae have been frequently reported as primary benzene degraders under different electron acceptor conditions (Vogt et al. 2011; Kleinsteuber et al. 2012). In our study, Peptococcaceae phylotypes were detected in low relative abundances (0.5–3.3%) in some of the communities enriched with carbonate, Fe(III) NTA or goethite as electron acceptors (Table SI 2); in the communities amended with Fe(III) oxyhydoxide which mineralized the highest amounts of benzene, their abundance was however below 0.5%. Further work is necessary to clearly identify the benzene mineralizing organisms in the enriched communities.

The sulfate amended cultures did not mineralize benzene but produced sulfide due to mineralization of background electron donors of the original sediment. Rhizobiales, Bacteroidales, Desulfarculales, Desulfobacterales and Clostridiales were the most abundant taxa in two analyzed microcosms. Interestingly, their community compositions differed completely although generating similar sulfide amounts; a possible explanation is that the grown organisms occurred in the original inoculum at small abundance and were randomly distributed during preparation of subcultures by aliquoting them. More than 50% of culture Sf2 comprised of typical sulfate reducing taxa (Desulfarculales, Desulfobacterales, Desulfuromonadales, Desulfovibrionales). Remarkably, culture Sf1 was made up to around 75% by phylotypes belonging to the Rhizobiales which is a taxa not known for sulfate reduction; typical sulfate-reducing taxa were absent except a few Desulfovibrionales. Strains belonging to the Rhizobiales have not been described for sulfate reduction. However, they were recently enriched from deep subsurface sediment using acetate and sulfate as electron donor and acceptor, respectively, indicating that some organisms belonging to this group may be able to thrive under sulfate-reducing conditions (Purkamo et al. 2017).

## Conclusion

This is the first report of anaerobic hydrocarbon degradation in the hydrocarbon-polluted Niger Delta sediments. The results demonstrated a natural attenuation potential of the investigated subsurface sediments for anaerobic hydrocarbon degradation especially under iron-reducing conditions, using benzene as model compound. The analysis of the microbial communities indicated that phylogenetically different organisms were involved in anaerobic benzene mineralization in the Niger Delta sediment samples. Betaproteobacteriales, Ignavibacteriales, and Desulfuromondales including Geobacter, which was previously observed as benzene degrader under iron-reducing conditions, were the most abundant phylotypes during benzene mineralization at methanogenic or iron-reducing conditions. In the cultures amended with amorphous Fe(III) oxyhydroxide that showed the highest mineralization rates, Desulfuromonadales were not enriched, hence Geobacter was very likely not the primary benzene degrader in these cultures. The dominant phylotypes included Betaproteobacteriales and Ignavibacteriales, which are the putative benzene degraders under these conditions. However, due to the relatively low amounts of mineralized benzene, we cannot exclude that organisms detected in minor abundances in the analyzed communities were actually involved in benzene degradation.

## Electronic supplementary material

Below is the link to the electronic supplementary material.

Supplementary file1 (DOCX 1291 KB)
