# Supplementary material for: Anaerobic benzene mineralization by natural microbial communities from Niger Delta
Source: Biodegradation. 2020 Dec 2;32(1):37–52. doi: 10.1007/s10532-020-09922-x (PMC7940306; doi:10.1007/s10532-020-09922-x)
Supplement: Supplementary file 1 — Supplementary file1 (DOCX 1291 KB) [file 10532_2020_9922_MOESM1_ESM.docx]

**Supplementary Information**

**Anaerobic benzene mineralization by natural microbial communities from Niger Delta**

Samuel C. Eziuzor, Matthias Schmidt and Carsten Vogt*

Department of Isotope Biogeochemistry

Helmholtz Centre for Environmental Research-UFZ, Permoserstraße 15, 04318 Leipzig, Germany

**Author information**

* Corresponding author: carsten.vogt@ufz.de

**Acknowledgments**

S.C.E. acknowledged funding from the Deutscher Akademischer Austausch Dienst, DAAD.

We thank Stephanie Hinke, Ute Lohse and Denny Popp for their technical assistance during cultivation, Illumina sequencing, and technical support in QIIME2 pipeline analytics, respectively. The authors are grateful for using the helium-ion microscope at ProVIS – Centre for Chemical Microscopy at the Helmholtz – Centre for Environmental Research, Leipzig, which is supported by European Regional Development Funds (EFRE – Europe funds Saxony), and the Helmholtz Association.

**Supporting Information – Table of content**

**S1 – Materials and Methods**

**S1** Calculation of mineralization rates

**S2** Scanning helium ion microscopy (HIM)

**S2 – Tables**

**Table S1** Rate of mineralization of fully labeled ^13^C-benzene used in the enrichment cultures under different terminal electron acceptor conditions

**S3 – Figures**

**Fig. S1** Scheme showing how the cultures were transferred at different iron-reducing conditions. For each transfer, 10% of the culture suspension were taken.

**Fig. S2** Microbial community compositions from the sediments obtained from the four administrative areas of Ogoniland, a microcosms of Niger Delta, Nigeria

**Fig. S3** HIM micrograph of non-filamentous and filamentous prokaryotes, and organic structures found in iron-reducing cultures with Fe (lll) NTA

**S1 Calculation of mineralization rates**

The rate of mineralization was calculated using the equation stated in Dorer et al. (2016) based on the following assumptions that;

1. carbon isotope fractionation effects of CO_2_ between aqueous and gaseous phase can be neglected (Winderl et al. 2010).
2. the CO_2_-pool remains constant during the mineralization process (the amount of CO_2_ in the beginning is much larger than the generated CO_2_ during mineralization ([CO_2_]^(start)^ >> [CO_2_]^(min)^)
3. the very small concentration of natural ^13^C in the biotic carbon pool can be neglected

For the calculations, the following parameters have to be known:

1. the concentration of the bicarbonate [HCO_3_^-^] buffer in the medium,
2. the initial ^13^C/^12^C ratio R_(start)_ (at time t_0_ after substrate addition) = ẟ_0_
3. the initial concentration of the partially or fully ^13^C-labeled substrate [substrate]
4. in case of partially ^13^C-labeled substrate the number of unlabeled C atoms (x) in comparison to the total number of C atoms of the substrate (n)
5. and the ^13^C/^12^C ratio R_t_ at a later time t = ẟ_end_

$$Rate of mineralization = \frac{[HCO_{3}^{-}]* R_{VPDB} * \left( \delta_{end}- \delta_{0} \right)}{\left( n-x \right)* \left[ substrate \right]*\{1+ R_{VPDB}* \left( 1 +\delta_{\left( start \right)} \right)\}}*100$$

**S2 Scanning helium ion microscopy (HIM)**

*Sample preparation*

Samples were taken at the end of experiment from enrichment cultures incubated at iron-reducing conditions using Fe (lll) NTA as iron source to investigate putative iron bacteria filaments structures using HIM. Samples were prepared following the protocol given by Said et al. (2018). Briefly, samples were fixed in 1 % paraformaldehyde solution prepared in sodium cacodylate buffer at pH 7.4 for 2 h at room temperature. Subsequently, aliquots were filtered through a polycarbonate filter (0.22 μm pore size), rinsed, post-fixed in 1% osmium tetroxide solution for 1 h (both also prepared in sodium cacodylate buffer), dehydrated in a graded ethanol series and critical point dried. Note that the samples were not sputter coated with a thin layer of metal as would have been necessary for scanning electron microscopy.

*Microscopy*

Imaging was performed with a Zeiss Orion NanoFab HIM (Carl Zeiss Microscopy, Peabody, MA) (Joens et al. 2013) in secondary electron detection mode using an Everhard-Thornley detector. The ion landing energy amounted to 25keV the beam current was approximately 0.8pA. Charge compensation was achieved by using an electron flood gun after the scan of each line.

**Table S1** Mineralization rates of fully labeled ^13^C-benzene by enrichment cultures under different electron acceptor conditions

| Sample ID | Main electron acceptor | ^13^C-benzene  (µM) | HCO_3_^-^  (µM) | δ^13^C_end_  (‰) | δ^13^C_0_  (‰) | Duration (days) | Mineralization (% of added benzene) | µM benzene mineralized | µM benzene day^-1^ |
| --- | --- | --- | --- | --- | --- | --- | --- | --- | --- |
| Sf1 | Sulfate | 440 | 30000 | -27.0 | -26.97 | 780 | b.d.l. | b.d.l. | b.d.l. |
| Sf2 | Sulfate | 434 | 30000 | -25.3 | -26.92 | 780 | b.d.l. | b.d.l. | b.d.l. |
| Sf3 | Sulfate | 478 | 30000 | -25.7 | -27.20 | 780 | b.d.l. | b.d.l. | b.d.l. |
| Sf4 | Sulfate | 394 | 30000 | -26.1 | -27.15 | 780 | b.d.l. | b.d.l. | b.d.l. |
| Mt1 | Carbonate | 289 | 30000 | 64.0 | -24.33 | 780 | 1.7 | 5.0 | 0.006 |
| Mt2 | Carbonate | 294 | 30000 | 45.9 | -24.39 | 780 | 1.4 | 4,0 | 0.005 |
| Mt3 | Carbonate | 310 | 30000 | 177.0 | -24.32 | 780 | 3.7 | 11.5 | 0.015 |
| Mt4 | Carbonate | 294 | 30000 | 141.2 | -24.24 | 780 | 3.2 | 9.4 | 0.012 |
| Fox1a-initial rate | Fe (lll) oxyhydroxide | 304 | 30000 | 349.9 | -25.43 | 272 | 7.0 | 21.4 | 0.079 |
| Fox1b-after first transfer | Fe (lll) oxyhydroxide | 288 | 30000 | 1853.6 | 349.9 | 42 | 21.5 | 61.9 | 1.474 |
| Fox1c-late phase  Fox2  Fox3 | Fe (lll) oxyhydroxide  Fe (lll) oxyhydroxide  Fe (lll) oxyhydroxide | 143  354  311 | 30000  30000  30000 | 2067.9  320.1  490.4 | 1853.6  -25.2  -25.5 | 466  780  780 | 2.9  5.6  9.5 | 4.2  19.7  29.4 | 0.009  0.025  0.038 |
| Fox4 | Fe (lll) oxyhydroxide | 376 | 30000 | 642.4 | -25.45 | 780 | 10.1 | 38.1 | 0.049 |
| Fox5 | Fe (lll) oxyhydroxide | 242 | 30000 | 1296.3 | -22.34 | 440 | 31.0 | 74.9 | 0.17 |
| Fox6 | Fe (lll) oxyhydroxide | 232 | 30000 | 929.8 | -21.68 | 440 | 23.3 | 54.0 | 0.123 |
| Fox7 | Fe (lll) oxyhydroxide | 232 | 30000 | 3712.4 | -43.25 | 440 | 94.2 | 218.1 | 0.496 |
| Fnt1 | Fe (lll) NTA | 312 | 30000 | -14.1 | -24.49 | 780 | 0.2 | 0.6 | 0.001 |
| Fnt2 | Fe (lll) NTA | 243 | 30000 | 184.8 | -24.30 | 780 | 4.9 | 11.9 | 0.015 |
| Fnt3 | Fe (lll) NTA | 351 | 30000 | 232.9 | --24.20 | 780 | 4.2 | 14.6 | 0.019 |
| Fn4 | Fe (lll) NTA | 308 | 30000 | 78.9 | -24.16 | 780 | 1.9 | 5.9 | 0.008 |
| Gt1 | Goethite | 152 | 30000 | 69.2 | -5.69 | 220 | 2.7 | 4.2 | 0.019 |
| Gt2 | Goethite | 205 | 30000 | 204.7 | -4.42 | 220 | 5.7 | 11.6 | 0.053 |
| Gt3 | Goethite | 178 | 30000 | 260.8 | -10.34 | 220 | 8.5 | 15.2 | 0.069 |

b.d.l. = below detection limit

**Table S2**: Abundances of phylotypes belonging the Peptococcaceae in the microcosms. In microcosm not listed, abundances were generally <0.5.

| Microcosm | Amount of mineralized benzene (see also Table S1) | Family and Genus | Relative abundance [%] |
| --- | --- | --- | --- |
| Sf2 | 0 | Peptococcaceae, uncultured | 2.2 |
| Mt1 | 5.0 | Peptococcaceae, Desulfosporosinus  Peptococcaceae, Candidatus Dichloromethanomonas | 3.2 |
| Fnt1 | 0.6 | Peptococcaceae, Desulfitobacterium | 0.7 |
| Fnt2 | 11.9 | Peptococcaceae, Desulfosporosinus  Peptococcaceae, Candidatus Dichloromethanomonas  Peptococcaceae, Desulfitobacterium | 1.9  0.8  0.5 |
| Gt1 | 4.2 | Peptococcaceae, Desulfosporosinus | 1.6 |


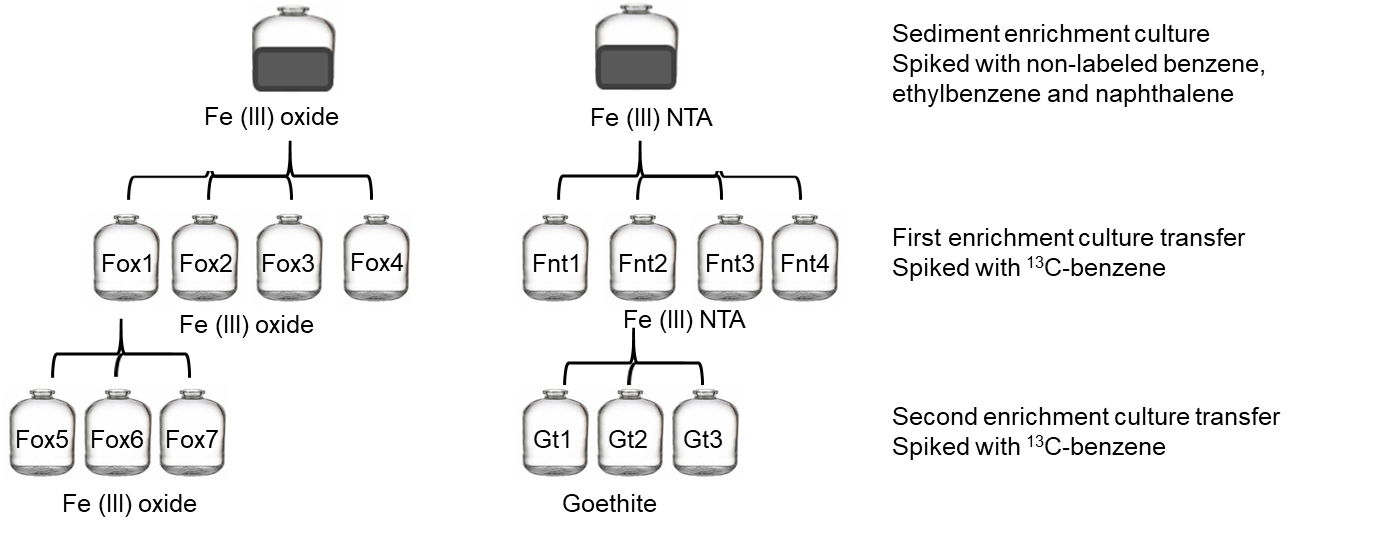


**Figure S1** Scheme showing details of the 10% enrichment culture transfer for iron-reducing conditions. The transferred cultures were selected based on their benzene mineralization rates.

**Figure S2** Microbial community compositions from the sediments obtained from the four administrative areas of Ogoniland, a microcosms of Niger Delta, Nigeria. The four areas in Ogoni from which sediments were obtained are represented as Gok (Gokana), Kan (Khana), Tai (Tai), and Elm (Eleme).


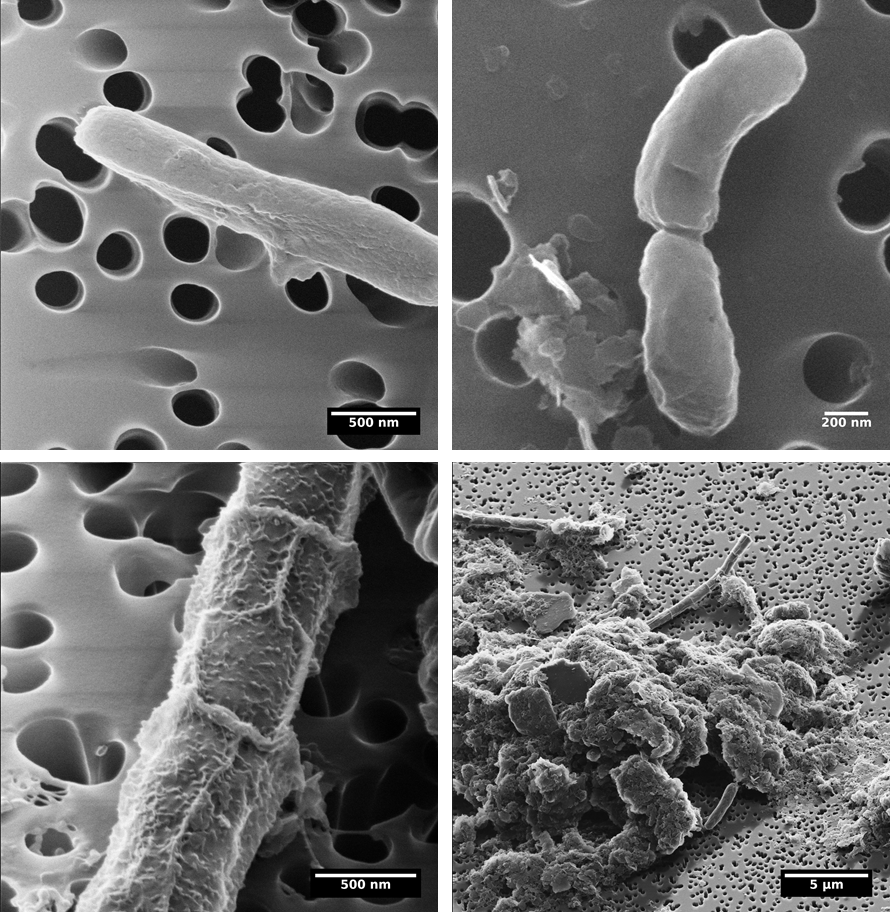


**Figure S3** Scanning helium-ion micrograph of non-filamentous and filamentous prokaryotes, and organic structures found in iron-reducing cultures amended with Fe(lll) NTA.

**References**

Byrne JM, Schmidt M, Gauger T, Bryce C, Kappler A (2018). Imaging organic-mineral aggregates formed by fe (ll)-oxidizing bacteria using Helium ion microscopy. Environ Sci Technol Lett 5(4): 209-213. [doi.org/10.1021/acs.estlett.8b00077](https://doi.org/10.1021/acs.estlett.8b00077)

Dorer C, Vogt C, Neu T, Stryhanyuk H, Richnow, HH (2016). Characterization of toluene and ethylbenzene degradation under nitrate-, iron (III)- and manganese(IV)-reducing conditions by compound-specific isotope analysis. Environ Poll 2111:271-281

Joens MS, Huynh C, Kasuboski JM, Ferranti D, Sigal YJ, Zeitvogel F. Obst M, Burkhardt CJ, Curran KP, Chalasani SH, Stern LA, Goetze B, Fitzpatrick JAJ (2013) Helium Ion Microscopy (HIM) for the imaging of biological samples at sub-nanometer resolution. Sci Rep 3: 3514.

Said N, Chatzinotas A, Schmidt M (2019) Have an ion on it: the life-cycle of *Bdellovibrio bacteriovorus* viewed by Helium-ion microscopy. *Adv Biosys* 3. DOI: https://10.1002/adbi.201800250

Winderl C, Penning H, von Netzer F, Meckenstock RU., Lueders T (2010) DNA-SIP identifies sulfate-reducing Clostridia as important toluene degraders in tar-oil-contaminated aquifer sediment. The ISME Journal 4:1314-1325
